# Speaking of Hunger: Food Shortages, Poverty and Community Assistance in Urban South Africa

**DOI:** 10.1080/03670244.2024.2361249

**Published:** 2024-06-02

**Authors:** Lucy Khofi, Lenore Manderson, Eileen Moyer

**Affiliations:** a School of Public Health, University of the Witwatersrand, Johannesburg, South Africa; b Department of Anthropology, University of Amsterdam, Amsterdam, The Netherlands; c School of Social Sciences, Monash University, Melbourne, Australia

**Keywords:** Food assistance, hunger, idioms of distress, poverty

## Abstract

How people speak of hunger extends beyond statements about food adequacy; people’s remarks may reflect experiences of poverty and feelings of vulnerability, and may be used to request help. In this article, we build on the idea of idioms of distress to conceptualize hunger talk as expressing more than an empty belly. We draw on ethnographic data gathered in two settings in South Africa: one a peri-urban area under traditional jurisdiction in the Eastern Cape Province; the other an inner-city suburb of the largest city, Johannesburg, in Gauteng. Hunger-related idioms of distress help illustrate the complex interplay of social, economic, and cultural factors, and allow people to speak of various affective and material aspects of their lives.

Experiencing hunger entails more than the embodied sense of an empty stomach. By analyzing how people speak of hunger, we aim to demonstrate the diverse challenges in people’s lives and how they address these. We argue that people articulate their hunger in various ways, transcending simple “I’m hungry” statements. In exploring this, we draw on ethnographic data from two sites, the kingdom located within King Williams Town to which we refer pseudonymously as Mqanduli, and in Lorentzville, Johannesburg. Informed by Mark Nichter’s foundational work on “idioms of distress,” we see statements of hunger as the means by which people discuss challenges in their daily lives, and deploy the idiom, in the absence of social connections and knowledge of places, to seek help. During observations and informal discussions at both research sites, people routinely remarked that they were hungry. As anthropologists, we noted unspoken expressions accompanying this statement, prompting us to delve deeper into the profound meanings behind expressions of hunger, so to gain a deeper understanding of discourses of lack of food. People who stated that they were hungry used this as plea for assistance and as a powerful expression related to poverty and social marginalization.

“Idioms of distress” are cultural expressions of suffering, whereby people express feeling unwell, sick, or in pain, or as ways to communicate other challenges, including those that are related to feeling disconnected or disturbed, or difficulties on economic wellbeing, interpersonal and other social relationships, and social roles (Mendenhall et al. 2019). Much of the literature, however, focuses on the use of idioms in medical and psychiatric contexts, and to indicate psychological distress (Mendenhall et al. 2019; Nichter 2010; Pedersen, Kienzler, and Gamarra 2010). In this article, we aim to expand the utility of this framework to explore links between food adequacy and social and economic marginalization. In doing so, we conceptualize the use of hunger as an idiom of distress which express more than and other than an empty stomach. Through this approach, statements of hunger reveal socioeconomic, social, infrastructural, and political factors that contribute to hunger’s various expressions (Anandi and Azis 2024; Mkhize et al. 2022; Ndhlovu 2024).

## Idioms of distress

Idioms of distress, as conceptualized by Mark Nichter (1981), are ways by which people speak of unease, revealing personal or cultural issues that might stem from conflicts, financial struggles, or clashes in beliefs. These expressions are acknowledged by families and communities as signposts of trouble, and appear to be particularly prevalent among people lacking extensive social connections (Desai and Chaturvedi 2017). When conventional methods of expressing distress fail, people resort to alternative ways, including physical symptoms, as adaptive responses to challenging situations (Desai and Chaturvedi 2017).

Nichter (2010) emphasized the dynamic nature of the context in which expressions of distress unfold, influenced by personal inclinations, family attitudes, and broader societal issues including political insecurity and violence. However, he did not take wider ecological issues into account. As he illustrates, expressions of distress evolve over time, blending old forms with new, with particular idioms gaining or losing significance. They manifest in the present, shaped by current life circumstances and societal changes, embracing emerging expressions of evolving coping mechanisms. Nichter (2010) contends that idioms of distress serve as a metaphor of stress or distress, evoking memories of past difficulties and shedding light on current stressors. States of distress, ranging from mild stress to profound suffering, impact people’s ability to participate actively within families and in the community. While some idioms help people cope by validating suffering, and allow others to acknowledge their distress, idioms may also signal mental health issues affecting both individual and community well-being, and so transcend normal everyday difficulties (Nichter 2010). We assert that idioms of distress may also be entangled with wider environmental stressors, such as drought in a farming community, or a lack of safe drinking water or cooking fuel in an urban setting. In the examples used in this literature, idioms of distress are largely used to capture individual disquiet, hence the relationship between idioms and mental health problems.

In discussing the complexities of idioms, Kirmayer and Young (1998) and Nichter (2010) argue that such idioms need not be regarded as strict and conventional expressions of distress. They contend that the meanings behind these expressions are often unclear and even contradictory. Nichter suggests that it is more beneficial to focus on how distress is displayed and negotiated rather than adhering to rigid interpretations; he proposes an anthropology that engages with the senses, which might bring together somatic and symbolic aspects of idioms of distress. In building this argument, he draws on Lee, Kleinman, and Kleinman (2007), and their work on the lived experiences of depression in non-Western societies, to caution against labeling emotional expressions as metaphors or idioms. Lee, Kleinman, and Kleinman (2007) argue that labeling can lead us to view these expressions as not genuine, while overemphasizing the (assumed) underlying meaning while minimizing the experiences of emotions. In extrapolating from this, we have focused on the statement “I’m hungry,” mindful that idioms of hunger may be both symbolic of social and economic distress, and literal.

Hence we argue that idioms of distress on hunger express more than (and as well as) an empty stomach. The Nutrition Equity Framework (NEF) developed by Nisbett and colleagues in 2022 highlights how social and political forces influence access to essential resources like food, healthcare, and support systems. They emphasize that unfairness, injustice and exclusion contribute to disparities in nutrition across different communities and generations, particularly impacting women (Nisbett et al. 2022), and stress the importance of addressing historical injustices and promoting redistribution to tackle these disparities.

In a South African context, this approach draws attention to the long arc of Apartheid, and its continued impacts, 30 years since its formal end, on the lives, livelihoods and life courses of Black South Africans. Mkhize, Mthembu, and Napier (2023) have illustrated this in their study of urban Mlazi in Durban, KwaZulu Natal, in which they draw attention to the financial constraints on Mlazi’s residents. Here the cost of food has risen, concomitant with changes in food production methods, which have been reshaped toward more industrial approaches and have altered people’s preferences in the types of food they want to consume (Mkhize, Mthembu, and Napier 2023). People who have moved from rural to urban areas, particularly to informal settlements, are most at risk of food shortages. A significant proportion of informal settlement households are female-headed, and women especially struggle with erratic opportunities for income, soaring food prices in local shops, and inadequate nutrition (Mkhize, Mthembu, and Napier 2023). Here and in our own study areas, as we describe below, idioms of hunger refer both to the absence of and/or desire for food, and to other goods and services, rights and power. Hunger is a metaphor of desire and need, not only a statement of physiological need.

Hunger is therefore both a biological phenomenon and a statement of neglect and, as we show below, at times a statement of desire to escape the constraints of such neglect (Bublitz et al. 2019; Delville and Norris 2017; Ndhlovu 2024). The two study areas in which we worked, as described below, were both marginal, their residents were subjected to a wide range of social determinants of hunger, including structural and systemic factors perpetuating food insecurity. The social determinants of hunger to which we refer include social, economic, cultural, and political factors that influence individual and community access to adequate and nutritious food, including production and distribution systems of food; employment, income and the affordability of food; and the domestic contexts in which food is (or is not) stored and prepared (Beaulieu and Blundell 2021; Nisbett et al. 2022; Nxusani, Zuma, and Mbhenyane 2023). But they include, too, the factors that underlie these variables: systems of monocropping, agribusinesses, supply chains, imports, exports and consequent pricing, globalization, and the economics of distribution. At times, as occurred during the harshest months of the COVID pandemic, these all reverberated at community levels and in households. The embodied feelings of hunger, expressed in the field, were often the end point.

## Setting

In this article, we draw on research conducted as part of a larger study concerned with water, energy and food in urban and periurban settings in South Africa (see Acknowledgments). The research was conducted in a residential and small-scale agricultural area on the outskirts of a large administrative center in the Eastern Cape, South Africa, whose residents identified as Xhosa and spoke isiXhosa. This area, which we refer to pseudonymously as Mqanduli, we consider peri-urban. The second study site was Lorentzville, in inner-city Johannesburg, the largest city in the country; the population was a diverse mix of immigrants from across South Africa and from other countries on the continent. Although the distinct local dynamics in the study settings suggest the need for a nuanced understanding within each community, there are striking similarities in the cultural expressions of people living in these sites. These underscore the wider significance of food and nourishment as a reflection of general wellbeing.

Mqanduli is referred to as the “Great Place” in reference to its status as the residential center of a Xhosa chief (referred to as a King), who continues to influence economic and political life in the region, and to some extent, along with other chiefs, at a provincial level. Further, while the traditional chief retains power and certain judicial authority in the area, not all residents come from this area or from this traditional authority, and they lack the rights that are linked to local patronage. Residents of Mqanduli, like those of other peri-urban areas, must navigate a complex socio-economic landscape as they balance traditional livelihood practices and the distribution of authority with contemporary systems and institutions of governance, and aspirations for economic development and social mobility. They must also manage uneven access to basic resources, including water, energy, and food. Not all houses have piped water or formal connection to electricity; variable opportunities and generally high unemployment rates result in low income; a significant number of students fail to complete secondary education, so reinforcing systemic educational inequities. Within this area, community-driven and other local initiatives have emerged, including gardening projects aimed at the cultivation of vegetables for sale as well as local tables. Some produce is grown locally with the support of development NPOs (not-for-profits), but there is not a large enough base to produce food for wholesale and not enough opportunities to provide a regular employment base or flow of income. Consequently, food is sold to retailers and to informal vendors.

Lorentzville in contrast has little land for gardening. It is a densely populated urban area, walking distance from the inner city and the opportunities this affords for income generation: minding parked cars for tips, waste collection and recycling, the sale of sex and drugs, begging, and scavenging. Despite a gentrifying business district that is relatively well-serviced by the municipality, residential infrastructure has been poorly maintained and neglected by the state, with apartment blocks in obvious need of repair, burnt out buildings in which people squat (in local terminology, which people “hijack”), and informal settlements where people have constructed shelter from cardboard, plastic, rusty sheets of corrugated iron, and other waste materials. Significant numbers of these residents lack recognized documentation – that is, no passport, no work permit or no study permit. They have limited entitlement to and poor access to basic services, including to grants from the government in the absence of paid work, and they live in an area characterized by high crime rates, endemic substance misuse, lack of employment opportunities, and entrenched poverty. Access to critical resources such as water, sanitation and energy is irregular, and food insecurity and felt hunger are simply the most spoken of these everyday hardships.

In both study sites, the majority of residents live below the poverty line, which includes most female-headed households, reflecting the gendered dimensions of economic hardship. By conducting our study in both peri-urban and urban settings, we sought to explore perspectives on the meanings of hunger beyond the direct and immediate experiences of the need for food. This comparative approach allowed us to identify commonalities and differences in individuals’ experiences across diverse settings regarding socio-economic disparities and the promotion of overall well-being.

## Methods

The data which we present emerged primarily from participatory and ethnographic research conducted by the first author, involving dynamic interaction with community members in the two study settings. In Mqanduli, (author) attended council meetings and observed participants working in the community garden, which contributed to a deep understanding of community dynamics. This immersive approach recognized participants as active citizens engaged in critical discussions related to gender, food security and food sovereignty. In addition to participant observation, data were gathered through informal discussions, formal interviewing, and focus group discussions. In Lorentzville, (author) employed a case study design, with active participation in local initiatives including assistance at a “soup kitchen” cooking food for immediate distribution, established soon after the pandemic was declared as people lost contract work. She also assisted in recycling initiatives; and in collaborative activities such as counting and packing donated food parcels for distribution to community beneficiaries of a local feeding programme.

In this article, we draw on data from 20 in-depth interviews and four focus groups conducted in Mqanduli, and 35 in-depth interviews and six focus groups in Lorentzville. Women comprised a higher representation within the sample, i.e., participants in Lorentzville included twenty-three women, ten men, and two participants who identified as other; in Mqanduli thirteen women, and seven men. Participants for interviews and focus groups were recruited using a combination of snowball sampling and purposeful sampling, ensuring diversity in terms of education and gender to account for a broad age range and diverse skills and activities within the communities. Interviews and focus groups were audio recorded and supplemented by notes; audio-tapes were subsequently transcribed (and sometimes translated from isiXhosa to English) for analysis. Transcribed text and fieldnotes were imported into a Word document for coding. A combined inductive and deductive approach to analysis was employed. Anticipated themes such as lack of access to food, water and energy were initially identified; additional themes, including those related to ideas of idioms of distress, emerged during consultation with supervisors. This comprehensive analysis allowed for the exploration of multifaceted issues related to water, energy, and food insecurity. Ethical clearance, with Ethics Number M220760, was obtained from the Human Research Ethics Committee (Medical) at the University of the Witwatersrand.

The dynamics of familiarity and acceptance of outside researchers varied. In the Eastern Cape, the first author’s positionality was that of an insider, to the extent that she had been born in the region (although not in the study area) and was fluent in isiXhosa. While she grew up in Johannesburg, her connection to the Eastern Cape allowed her easy rapport, and her status as a visitor facilitated access. Conversely, in Lorentzville, her unfamiliarity with the local population and their diverse backgrounds posed challenges. She was always accompanied by a community volunteer because of local concern regarding elevated crime rates in the area, and because of her limited proficiency in the dominant language (Sepedi, widely spoken in northeast South Africa). As she became more involved and contributed to community activities to address immediate food shortages, she was increasingly comfortable in the community and arguably gained acceptance from local residents.

## Findings

### Hunger in mqanduli

In Mqanduli, the community operates within a traditional political structure based on patriarchy and inherited leadership. Decision-making involves community gatherings under the king’s guidance, providing a platform for more men than women to voice concerns related to local governance, resource allocation and community development. These gatherings, occurring once a month, address the evolving needs of the community dominated by the King and those around him. (First author) worked closely with Mam’Qwathi, a female community worker from a different region in the province who had approached the king to focus on empowering community members to establish long-term food sovereignty. People live in small houses, some of which were built under the Reconstruction and Development Programme initiated in 1994 to provide shelter post-apartheid; other houses include rondavels. Roads are tarred near the King’s place, and in this area too, houses have access to piped water and an official supply of electricity. Elsewhere the roads are graveled and characteristically pot-holed, and people have limited access to water, sanitation, and energy. The local municipality, the Department of Agriculture, agricultural NGOs, and a nearby Technical and Vocational Education and Training (TVET) College, have all facilitated collaborative agricultural initiatives, including community gardens and education programs, as part of a concerted if uncoordinated effort to improve living standards. Conventional gender roles persist, with women predominantly handling household duties, i.e., cooking. However, both men and women worked in the community garden and associated sales, and a woman led the entire community initiative but was subjected to hostility as an outsider.

Multiple terms in the isiXhosa language are used in discourse on hunger: *ndilambile* (“feeling hungry,” but in a different context it can also mean “I’m tired”), but also *sihluphekile* (poverty), *sithwele kanzima* (we are burdened), and *silahliwe* (we are neglected). These terms were used in contexts referring to lack of food, but their choice suggested that people were referring to more than just the physical sensation of an empty stomach: each term incorporates a broader spectrum of desires and distress, longings and constraints, and the fundamental need for sustenance.

Ensuring successful cultivation and abundant harvests, preparing and sharing meals, and celebrating with food, are central to isiXhosa cultural practices and beliefs, underscoring the significance of nourishment (Ndhlovu 2024). Women are largely responsible for production or purchasing and preparing food, for both the household and community purposes. In isiXhosa folklore and oral traditions, narratives portray the ability of people to endure hunger as a sign of resilience, other narratives provide accounts of people sharing, endurance, and the pursuit of sustenance amidst adversity (Ndhlovu 2024; Nxusani, Zuma, and Mbhenyane 2023). But hunger is also a metaphor in contemporary settings. In our research, participants referred to being hungry for opportunities; hungry for education; hungry because they lacked electricity, piped water, and sanitation; hungry because they didn’t have access to basic needs (i.e. food, water, energy); and other socio-economic factors. A young participant, Sibonele said, “*Kubuhlung’ ukuphila apha* (it’s hard to survive here). We always fight to get access to government services. As you see, we don’t have electricity. Since last month, our communal taps have not been working. *Kanzima dadewethu* (it’s hard, my sister). Our people are hungry. *Ikat’ ilele eziko* (a Xhosa proverb meaning poverty). We don’t have job opportunities. We don’t have opportunities to go to universities. It is democracy but *asiyiboni inkululeko apha* (we don’t see democracy freedom here).”

During (first author’s) fieldwork in Mqanduli, women regularly spoke of hunger, stating “I’m hungry” or “we are hungry” in household interviews, often immediately after greetings and in response to the conventional enquiry into wellbeing or in response to inquiries about the local area. In addition, people often employed everyday expressions to communicate the challenges they faced, to depict the complex emotions associated with food insecurity. For instance, some would say, “Our stomachs are singing the song of hunger,” to describe their intense hunger and the struggles they were facing.

When (author) first entered the grounds of the Great Place, she was taken to the commercial greenhouse tunnels where residents were growing market vegetables. One young man introduced himself by stating *silambile kulendawo* (“we are hungry in this area”). Since it was her first visit to observe and see the site, (author) did not pursue this, but when she returned, again the same comment – “we are hungry here”– was made by different people. In an informal interview, Zenzele explained: *“Siyahlupheka kulendawo* (we are poor). We need all the help we can get; it is good to see people visiting us so that we can identify who can further assist us. With such initiatives, we need more help.”

In response to (author)’s enquiry about the extent of hunger, no interviewees reported that they had gone to bed without food in the past month. The expression of hunger conveyed not the absolute lack of food in households, but that interviewees had not achieved their profit goals and that the community garden had not performed as well as they had hoped. Their businesses, rather than their bodies, were hungry for more. Interlocutors further mentioned that, as they were still expanding the business, it was crucial for them to consistently reach their profit targets, ensuring an ample and regular supply of food to markets. One older woman, Mam’Nonceba, added:

This hunger is not only affecting us, but our local markets also did not receive more fresh produce from us; hence, everyone felt the hunger. When we produce more vegetables, our customers get an opportunity to access more fresh produce at an affordable price. Even big retail markets get a chance to stock locally, paying less than ordering bulk produce from other providers or distant towns within the Eastern Cape. We have supermarkets that rely on our produce, including street vendors and public markets that depend on us.

People stated that when they produced more vegetables, everyone benefitted. Hunger was therefore a shortfall in production that affected their markets and consumers, and thus, everyone who bought their produce had been hungry in the past month. As Sihle reiterated: “For the past month, we have been hungry, *sisi* (my sister). It has been challenging as we did not have enough produce, and as a result, we did not make enough profits.” More women than men were working in the Mqanduli Kingdom community garden, hunger related to poor profits, particularly in comparison to profits that they had generated in prior months. Ntando explained that as a result, they had even skipped their monthly celebration – a gathering, with meat cooked on a braai (barbecued) and shared, which all attended to rejoice in their production and increased profits. As Nonhlanhla explained: “The entire team is motivated when we put in extra effort, and these small celebrations encourage us.”

(Author) had been introduced as working on a research project supported by an international collaboration, and the woman leading the commercial food production initiative would joke and say, “Will we get funding for this project?” Other participants saw any outsider as a potential funder, and regular spoke of hunger as an argument for more resources to support the community garden initiative. References to hunger were a request for resources from anyone visiting the Mqanduli Kingdom site, and mention of the funded research project by the author raised (false) expectations. These expectations diminished over time, and instead, (Author) was asked to help community members draft business proposals to send funding requests to relevant agencies. Participants started to speak directly of their struggles with limited resources, and elaborated on the extent to which they relied on monthly government social security payments, such as pension grants, child care grants, and orphan grants (SASSA 2023). Nonhlanhla explained that they were “fortunate to be part of this initiative because it provides us with income, and *sizibona singosomashishini* (we see ourselves as business people). We take all the profit we generate, which motivates us to work extremely hard to expand our profits by producing more vegetables.” Participants involved in the community garden often emphasized that they saw themselves as entrepreneurs because they worked to generate profits from sales, and idioms of hunger emerged as a legitimate way to ask for money. Some participants would go as far as saying, “We need a constant flow of money to expand our profits,” which they saw as the purpose of being involved in business and entrepreneurship.

A different expression of hunger occurred during interviews and home visits, when people would comment: “We have been hungry in this land.” One elderly woman, Mam’Nxesibe, explained:

*Mtanami* (my child), we have been struggling in this place for far too long, with no change since we were promised *inkululeko* (democratic freedom). Our people have been neglected and remain impoverished throughout our ancestral land. The Eastern Cape, at large, has been facing numerous challenges, including poverty, for centuries, *Mtanami*. Our children lack access to education and other opportunities, and we solely rely on social grants, which are not sufficient. As elders, we depend on pension social grants, which are less than R2090 (c.US140) per month.

Zikode made a similar point:

I was born post-1994, and I expected to see a difference in the empowerment and development of young people, but our government is not doing anything about this. We remain neglected and hungry. Many things, such as infrastructure, are still a big challenge in this community. We find many people drowning their lives in substance use, especially alcohol.

Other participants likewise argued that the state was not fulfilling its obligations or responsibilities. People argued that the government had failed to ensure that people had access to jobs, that it had not helped young people to be empowered and had neither enabled them to starting their own companies nor gain the relevant skills for employment. Women especially therefore applied for grants so that young people could apply for seed funding to be entrepreneurs:

Yes, *siyahlupheka* (we are suffering)! Yes, we are hungry, but we do not want handouts – we do not want social grants only, we need funding that funds certain business concepts. So many people have brilliant ideas but because of lack of funding, they cannot implement their ideas. (Baninzi)

Some participants spoke of hunger too in relation to exclusion, when they noted that not all community members were able to participate in the kingdom’s agriculture initiatives. They felt that this was not fair – all of them needed (“were hungry for”) an income. Participants started questioning the selection model, stating that people closest to the ruling family (whether friends or family) were most likely to have an opportunity to be part of the project. Others appreciated what the kingdom was doing and highlighted that the government could come on board by providing more resources to assist more community members.

We are blessed that we have the kingdom, and they can collaborate with the government – in some communities, it is worse, *sisi* (sister). If you look a street away from this house (near to the royal residence), there is no tar road, it is a gravel road. Why? It is because my great-grandparents built next to the kingdom and had an opportunity to access some of the resources just like that. In this street, we never run out of water in comparison to other streets and neighborhoods (Linamandla).

Other participants emphasized that living in an area far away from the largest towns in the province, such as East London or Gqeberha (Port Elizabeth), made it especially hard, and that they were deprived of opportunities that were primarily available in these towns:

People living in such areas are hungry and we are not given opportunities; we are not getting attention such as people do in big cities, like Port Elizabeth or East London. Even though we are still under the kingdom leadership, which is supposed to be prioritized according to traditional customs. During our great-grandparents’ time in the early 1900s, kingdoms were prioritized by the government, but now we have to fight harder to survive and try side gigs, like opening a tuck shop or selling second-hand clothes in town. *Kunzima ukuba apha sisi*. (It’s difficult living here, my sister) (Nozipho)

Other young people emphasized that they were hungry for education. They believed that education could change their lives and take them out of poverty and the social and economic structures that produce hunger:

As young people, we are not given opportunities, such as going on to further our education. When one wants to further their studies and apply for higher education, like (the University of) Fort Hare, Nelson Mandela University, or Walter Sisulu University, we do not get funding, as much as we are told that students living below a household income of R350 000 qualify for (government) funding. When we apply, we still do not get the funding. I believe that education can take me out of hunger and poverty, but I’m deprived of that opportunity. (Sibonelo)

Young people spoke of their desire to access higher education, using hunger as a term both to represent aspiration and motivation, and to refer to the costs of failed hopes. Older people expressed concern that their children were hungry but did not have opportunities. They too highlighted the lack of job opportunities and funding for young people to further their studies: “Even Technikons or colleges do not accept our children; they demand money. It is challenging to find a school that one can afford, as we do not have any money. Education is expensive, and *siphuma endaweni ezihlwempu* (we come from impoverished areas)” (Nomzekelo).

The shared sentiments of community members underscore the ongoing struggles for education and opportunities in the face of rising food prices, fluctuations in production and markets, literal hunger, and poverty. Financial constraints, unmet promises of funding, and a lack of accessible educational institutions contribute to the challenges faced by both young and older people. The narratives from research participants reflect the desire for education as a pathway out of poverty, emphasizing the need for improved educational support and opportunities in these communities. They also used “hunger” when expressing their concern about income, and so the capacity to purchase sufficient food; such hunger was often presented as a risk rather than a present, literal experience.

### Idioms of distress in lorentzville

Lorentzville is an inner city area of Johannesburg, primarily residential but with a small mix of business offices and warehouses as well. The population is dense, including immigrants from across South Africa. However the majority of residents in Lorentzville are foreign nationals, many without work or school permits, or the residential permits that are required for employment in various jobs or, in the absence of wages, for access to state support via monthly benefits.

For instance, Najma is a qualified social worker from the Ivory Coast, but because she does not have a working permit, she does not have a job. She survives by doing volunteer work (for which she might receive small compensation payments) and by selling secondhand clothes on the streets. Many participants had a similar profile. Onko from Nigeria is a qualified engineer, but because he does not have a work permit and his passport expired five years ago, he cannot look for employment aligned with his qualifications. Diallo from Senegal has a diploma in business and information technology, but he also could not gain formal employment as he did not have a passport or a work permit. Unemployment is extensive, and people face multiple socio-economic and material challenges, including lack of food, water and energy. Many people live and work on the streets, and even the most informal opportunities for money making reduced dramatically during the harshest weeks of lockdown (Manderson and Levine 2021).

The first use of what we see as idioms of distress was when participants mentioned that they were living in a neglected place, and that it had been like that for ages. Zelda said: “My family and I live in that building where there are no windows. We are always hungry – we do not have any opportunities, and it’s hard to survive here.” Another participant explained:

I moved into this location 15 years ago, and even now, I still do not have any documentation like a work permit and my passport expired long ago. It is so hard to be a foreign national. When we need help from the government, we do not get access to such help because we are illegal in the county. Our children are struggling; some are not even attending school, and this will have an impact on their future. Since this month, we have not had a decent meal. (Asante)

As noted earlier, in this area people live in crowded, substandard dwellings, including in burnt-out and deserted buildings and shacks, and they often occupy houses without title deeds. Many lack official papers as residents, and they experience considerable xenophobia that impacts their opportunities to find work. Participants consistently argued that the area was neglected, and they were hungry or had not had a decent meal or real meal. Carol, from Nigeria, explained:

It has been years without having a decent meal. My household appreciates small things; as long as we have maize meal, cooking oil, a tin of fish, or any vegetables, we are content. However, we have not had a decent meal, which includes meat and other treats like fruit juice and milk. I grew up in a household in Nigeria where a plate was not complete without meat. For most of my African friends, a meal is not enough without meat.

Other participants echoed this sentiment by stating that a plate of food in Africa is not complete without meat. Aboma said: “In Africa, we eat meat. It becomes very difficult when we are starving our children, giving them unbalanced meals. We grew up eating meat.” Throughout the interviews, South Africans from Johannesburg and other provinces – Limpopo, Eastern Cape, and KwaZulu Natal – as well as immigrants emphasized that a plate without meat was not “decent.” By speaking of hunger, people emphasized that they were referring to the absence of meat in their diet. A meal without meat was not balanced.

Informal income opportunities largely collapsed during the harshest weeks of first the COVID lockdown in Johannesburg, leading people to increasingly rely on community-based organizations and churches. Initiatives to address the living conditions of people in Lorentzville were established, or expanded, with donations from private businesses and nonprofit organizations to survive. On a daily basis, people faced the literal dilemma of empty cupboards, and many in the community turned to soup kitchens, community gardens, recycled waste and discarded food items. In this context, nonprofit organizations began to provide temporary hunger relief in the local community.

A year after the beginning of the pandemic and its harshest lockdowns, asked how they accessed food and what kind of food was available, participants stated that sometimes they still ate in community kitchens around Lorentzville. Although they emphasized that these kitchens did not serve a balanced or “decent” meal, they ate there because they were hungry:

At times, they literally give us pap (maize meal) and vegetable soup. This vegetable soup contains carrots, green pepper, salt, potatoes, and sometimes there is no cooking oil; they just boil the soup, and this can be untasty at times. But we eat since we are hungry; remember, beggars are not choosers. We eat what is available. I don’t remember the last time I bought proper groceries in a grocery shop or retail shop. When I happened to have money because of the sweets I sold on the streets, we bought from the spaza shop. (informal shop) (Linda)

Many participants explained that they were unable to buy food from large grocery shops because food items were much more expensive there, while spaza shops allowed them to purchase small quantities of a limited selection of foods, such as maize meal, tinned fish, vegetables, cooking oil and soup. Most could not afford meat and various participants recounted that sometimes they became ill after eating food items purchased from these shops:

(We buy from them) even though these shops sell us expired food items. There are times when we get sick, such as a runny stomach, feeling pain in the stomach, and vomiting when we eat some food items. (People from) a certain political party came to Lorentzville to check the expiry date of food items sold in these shops, and they found more expired items. They warned us not to buy in these shops, and two shops were closed, but we still buy there because it is what we can afford. (Oray)

While participants in Mqanduli used hunger as an idiom of aspiration, in Lorentzville, people were more likely to speak literally. (Author)’s fieldnotes bring this into sharp focus:

As we stepped into one shack on a scorching sunny day, we saw three children, aged between 5 and 13, lying on the floor; two older women occupied a small bed. Upon our arrival, the children rose, saying “We are hungry.” It seemed like they were waiting for the moment for a visitor to enter. One woman stood up and apologized, and chased the children outside. Before the volunteer could introduce us, she said: “Before we talk, can you please help us with bread? The children have not eaten the whole day today, and it is late afternoon.” The volunteer said, “It’s okay; I will run and buy bread.” The woman said, “I meant, what we will eat later as well?”

In this example, “bread” referred not only to loaves of bread, but to any food that might be eaten that day. The volunteer went to the warehouse of the local soup kitchen, and came back with food items that could last them at least a week. Other participants also asked for bread, but when provided with bread, they explained that they meant enough food to see them – their household – through the day. Where possible, (Author) and the volunteer referred these families to the warehouse, although food items were often in limited supply there as well.

But people also used hunger as a more general request, and like people in Mqanduli, they linked it to social structure and promises of political change. Some suggested that hunger strikes the unseen, people whose suffering is invisible. “When we talk openly about being hungry – it means we are now crying for help,” Marjorie explained. “We used to pretend that we were okay and hid our pain, but the community is now fed up, and we are crying for help, looking for help from NGOs, the government, and civil society at large” (Mahumba). Participants emphasized the urgency of getting the help they needed. Hunger caused considerable stress:

I’m not okay, the family is not okay. Living in such conditions where we are neglected, every day one must hustle for a plate of food. Sometimes I feel like I’m overthinking, but this is my reality and I have to deal with it, especially as a man in the house. How can one survive in such conditions? Surely something can be done for these conditions we are facing to be prioritized. (Bethuel)

People spoke of not feeling well: they felt drained, like there was no purpose in life, and they were always battling to survive. They reiterated that they were tired of being hungry and having empty plates. One participant gave the example of a clock ticking, and they were still hungry. It had been a long time without help, he explained, and now it’s time to seek help.

The phrase “the ticking of clocks on empty plates” was used by participants during household interviews in Lorentzville where individuals conveyed their hunger while discussing various aspects of daily life. This saying seemed to underscore the imperative for action in the face of hunger, and to encapsulate the emotional strain associated with deprivation. The larger metaphor also emphasized the cumulative impact of never having access to sufficient, essential resources – money, work, shelter, or food – adding urgency to requests for timely assistance.

In Lorentzville, residents voiced concerns over stark disparities in living conditions, highlighting feelings of neglect by the state. Basic services like community cleaning were notably absent, and streets were often littered with waste. The sense of abandonment was particularly acute among immigrants lacking proper documentation, who therefore lacked access to social grants available to South African citizens and legal residents, and who found it especially hard to find paid work.

## Discussion

Hunger is more than its physiological dimensions; it encapsulates a sense of neglect within marginalized populations, reflecting systemic issues of social inequality and deprivation (Nisbett et al. 2022). Our findings highlight the symbolic layers of hunger discourse, and the implications of this in understanding the socio-economic realities of poor people in both study sites. Here marginalized communities are subject to systemic injustices (Farmer 2004) that contribute to food insecurity.

In the stories from people in both settings, hunger always features as a concern. People spoke about times when they could not afford meals, when they had to rely on government assistance, and when they faced difficulties meeting daily needs. In Mqanduli, people spoke of hunger as aspiration and as a sign of failure; in Lorentzville, participants expressed hunger as neglect by the government to meet their basic needs. The interviews highlighted local inequality in accessing food, and the prevalence of food insecurities, but also of other shortages, deprivation and lack of options. People spoke of how much food was available, how easy or not it was to get, how it was used, and its overall stability, control and sustainability in the communities. The stories paint a stark picture of everyday difficulties.

The exploration of idioms of distress in Mqanduli and Lorentzville revealed that the phrase “we are hungry” was sometimes literal, but it was also a means of articulating poverty and resource scarcity. In the following, we review these expressions of distress, and the hidden language, codes, and ways of speaking hunger.

Women and men in both Mqanduli and Lorentzville emphasized their experience of physical hunger, signifying immediate or regular lack of food. However, as we have illustrated, the challenges extended to not being able to purchase groceries from a store, and relying on local spaza shops only when they had cash in hand. In Lorentzville, unemployment, especially among foreign nationals, compelled some to resort to informal means of earning, such as selling drugs, sex, or secondhand clothes. They also accepted food from volunteer kitchens, although at these availability was inconsistent and not all opened regularly. In addition, men were reluctant to collect food items directly, opting to send their children instead, citing concerns about preserving their dignity. In contrast, men and women in Mqanduli took pride in working on the community garden project, likely because they were entitled to all the profits made. For them, “hunger” was a metaphor for low profit, against their own aspirations. But they also faced limitations in relation to the unequal distribution of benefits from community gardens and challenges accessing local supermarkets.

Another expression of hunger manifested as a feeling of neglect and a plea for support and assistance. In Mqanduli, participants reflected on persisting challenges post-apartheid, including limited access to opportunities, education, healthcare and basic services. Disparities in infrastructure fueled their ongoing hunger or yearning for equal opportunities. Participants held the state responsible for not fulfilling its constitutional obligation to provide essential elements for a decent life, encompassing healthcare, food and social security. Likewise, in Lorentzville, people saw the state as failing to meet their needs as residents, whether or not they were formally so entitled under rules relating to citizenship.

Beyond the physical implications, participants associated hunger with psychological impacts, including stress, feeling overwhelmed, and a sense of purposelessness, although also, when speaking of hunger aspirationally, as a sense of purpose. Expressions of hunger largely highlight the multifaceted consequences of lack – of resources, money or food – but also desire. Mqanduli, participants expressed a hunger for education, citing fewer opportunities compared to East London and Port Elizabeth, the two largest cities in the province. Regardless of gender, individuals share a common aspiration for education. This suggests that educational attainment is a universal aspiration, transcending gender norms. The hunger idiom reflects a collective longing for education as a means to achieve broader aspirations, highlighting its significance as a unifying factor in shaping aspirations.

Hunger – inadequate or insufficient food – remains a pressing issue in South Africa, with many households facing challenges in accessing sufficient and nutritious food. The COVID-19 lockdown exacerbated these issues, with a notable increase in households running out of money for food (van der Berg, Patel, and Bridgman 2022). Similarly, droughts, water shortages and energy shortages have worsened hunger. In Mqanduli, residents heavily depended on social grants for survival, hence their “hunger” for profits from the community gardens. In Lorentzville, challenges intensified for participants who did not qualify for social grants due to their migrant status.

## Conclusion

In our exploration of idioms of distress, we focused on the diverse ways people express hunger, recognizing it not just as a physical need, but also as a powerful symbol of neglect and, in contradiction, also as a statement of aspiration. The varied expressions of hunger in the peri-urban setting of Mqanduli and the urban inner city area of Lorentzville illuminate the complexity of food insecurity at individual and household levels. The idioms of distress articulated by community members go beyond a simple acknowledgment of lack of food and its felt effect as hunger; they signify intricate webs of economic, social and psychological challenges that underscore the depth of their struggles.

Participants in Mqanduli reflected on the lingering disparities in wealth, opportunity, and access to services, relating the chronicity of societal neglect despite constitutional promises. Their plea for assistance was vocalized in the metaphoric request to “buy us bread” for support beyond immediate sustenance. The agricultural context in Mqanduli introduced yet another layer, whereby hunger was not merely a personal experience, but a collective concern tied to the success of community projects and their impact on local markets.

In Lorentzville, culturally specific expressions of a “decent meal” underscore the interconnectedness of food and identity. People’s inability to afford culturally significant foods, coupled with health risks associated with the consumption of expired items, highlighted the socio-economic constraints that exacerbate hunger-related challenges. Beyond the physical, the psychological toll of hunger was evident in expressions of stress, feeling overwhelmed, and a sense of purposelessness. These dimensions added depth to our understanding of the impact of food insecurity on individuals’ overall well-being.

Expressions of distress are context specific, and are shaped by unique cultural, historical, and socio-economic factors. Structural neglect and poverty in both settings exacerbated food insecurity, and the ways that women and men see to escape such constraints. The physical manifestations of hunger, as revealed through limited access to food and reliance on informal means of earning, paint a vivid picture of the daily struggles of residents in these communities.

Hunger emerges not only as a biological need but as a symbol of neglect and a call for intervention. In navigating the complexities of hunger, collaborative efforts are needed to tackle systemic issues, such as unemployment, disparities in access to resources, and broader socio-economic inequalities. This will involve the shared effort of policymakers, NGOs and communities, and the need to go beyond small community gardens and community kitchens. By addressing the underlying conditions that contribute to food insecurity, we can strive toward a more equitable and resilient future where the language of hunger is replaced with a collective commitment to ensuring food security for all.
